# Supplementary figures and images for: Bayesian hierarchical models for disease mapping applied to contagious pathologies
Source: PLoS One. 2021 Jan 13;16(1):e0222898. doi: 10.1371/journal.pone.0222898 (PMC7806170; doi:10.1371/journal.pone.0222898)

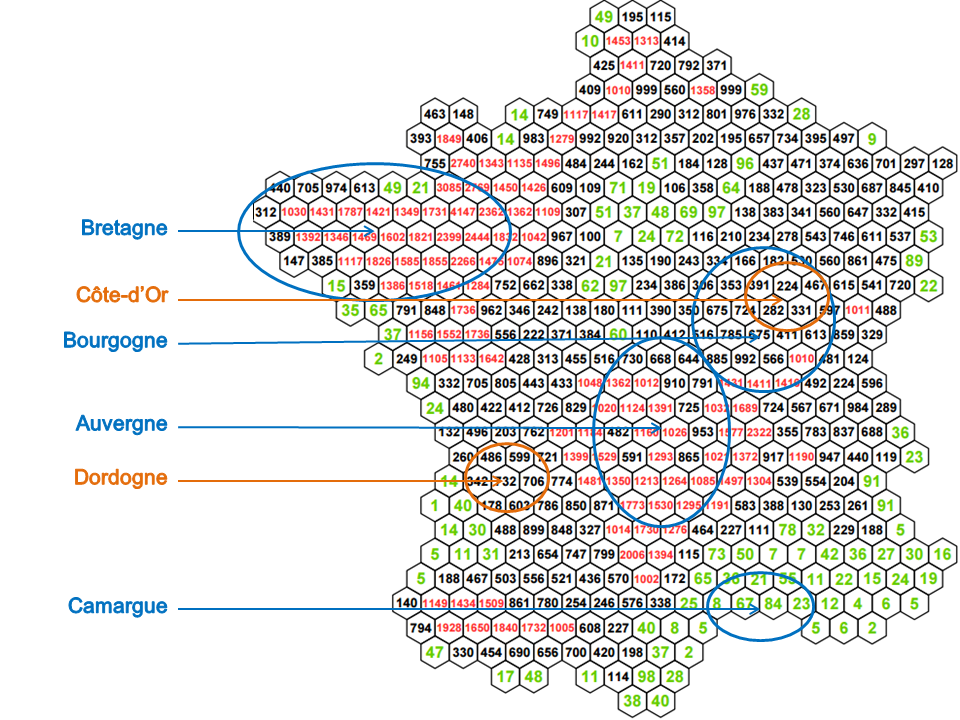

Supplement: S1 Fig — (TIFF) [file pone.0222898.s001.tiff]

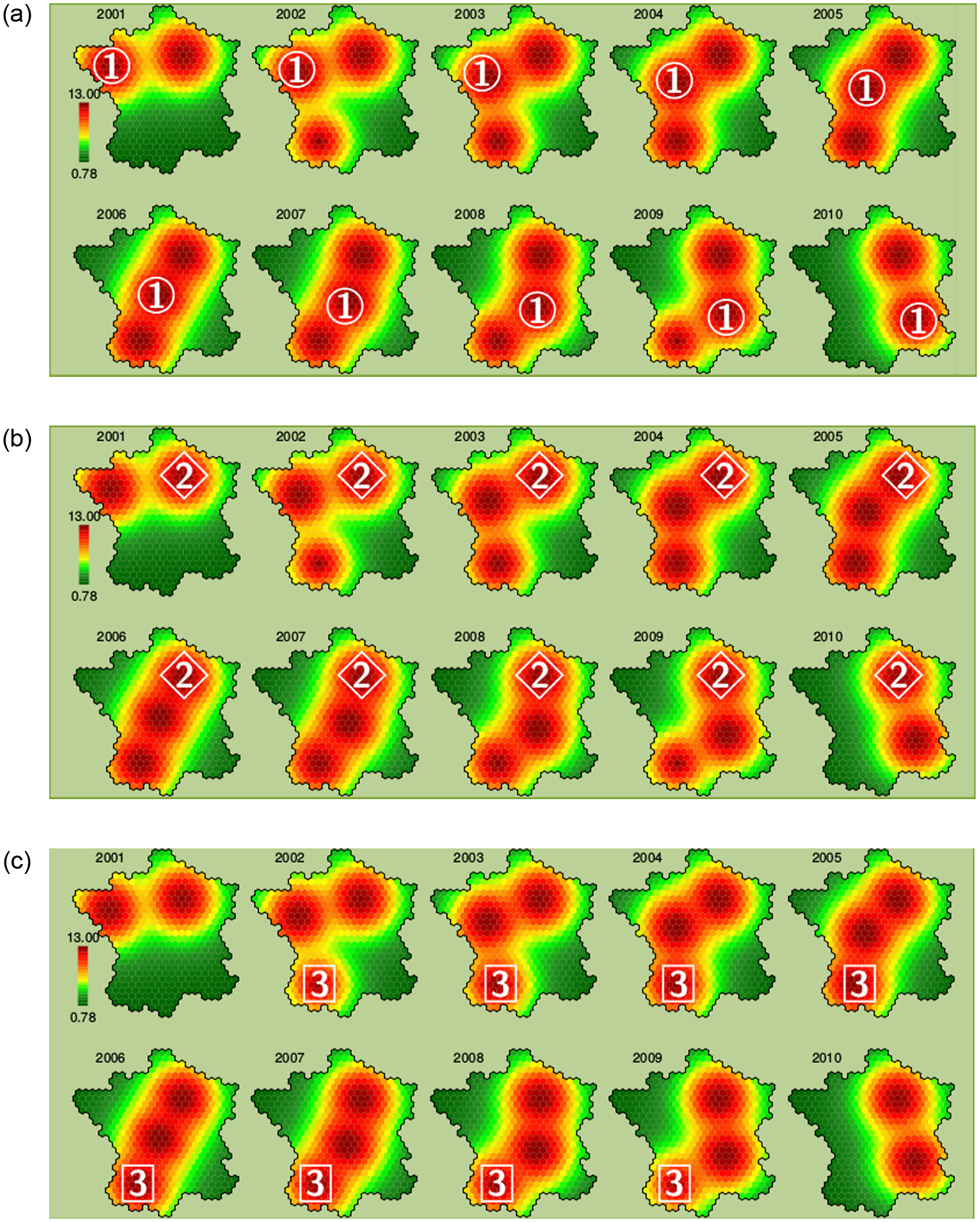

Supplement: S2 Fig — (TIF) [file pone.0222898.s002.tif]

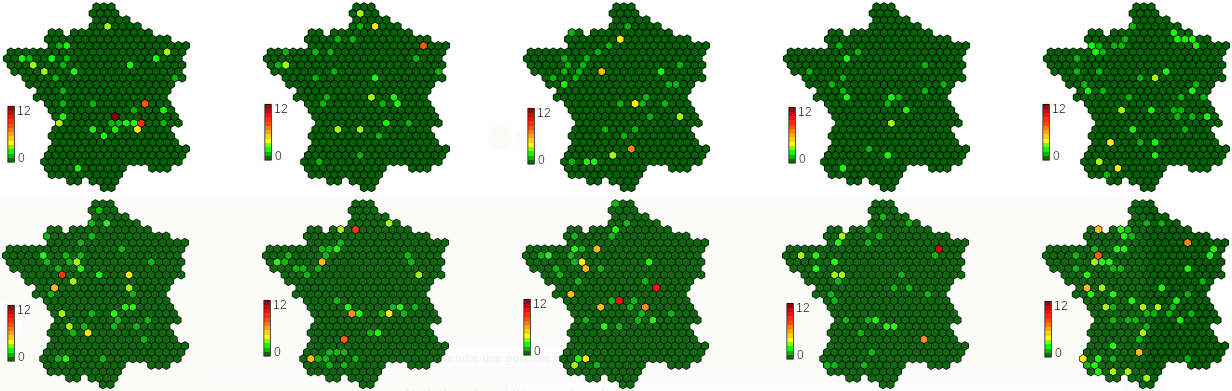

Supplement: S3 Fig — (TIF) [file pone.0222898.s003.tif]

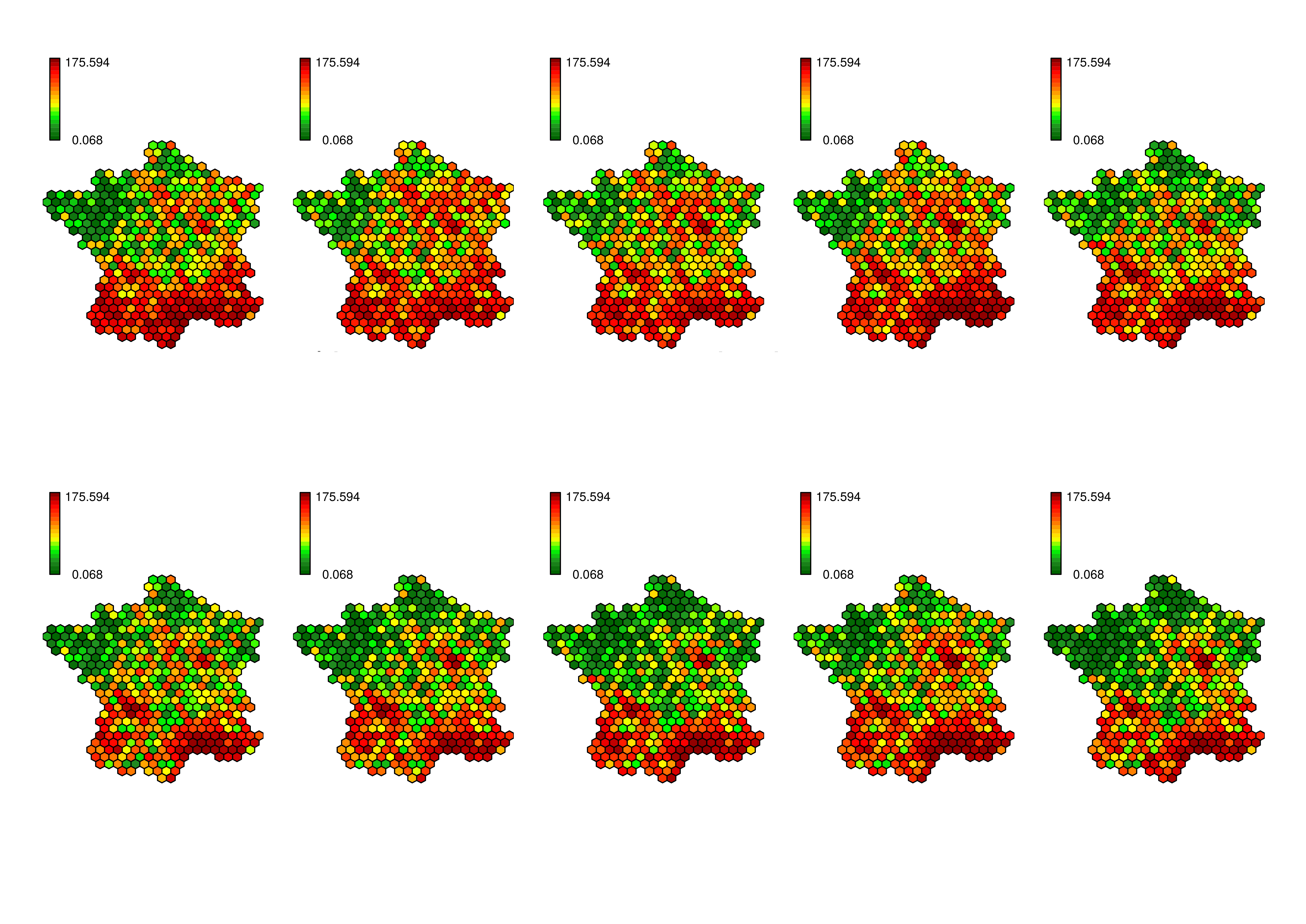

Supplement: S4 Fig — (TIFF) [file pone.0222898.s004.tiff]

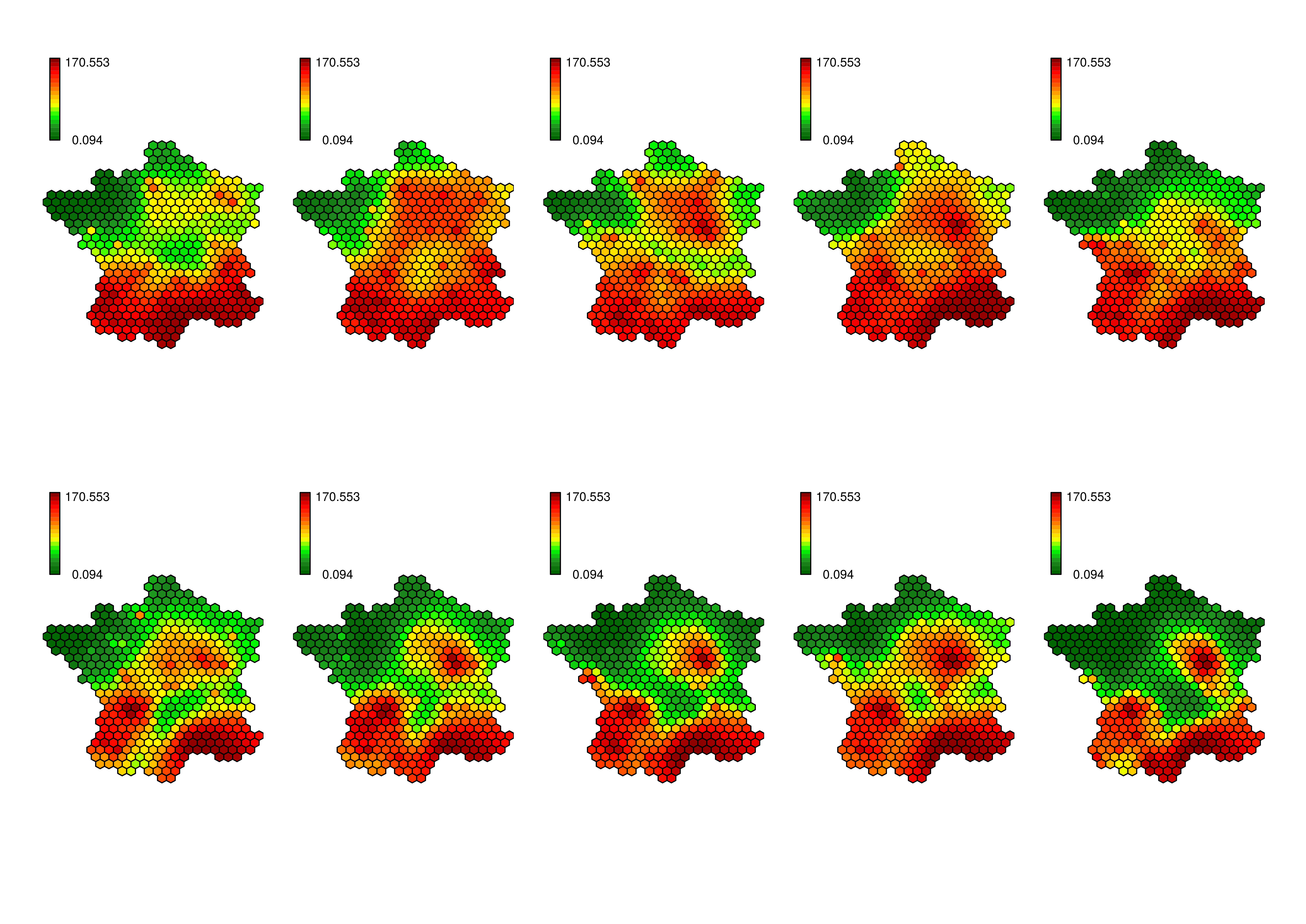

Supplement: S5 Fig — (TIFF) [file pone.0222898.s005.tiff]

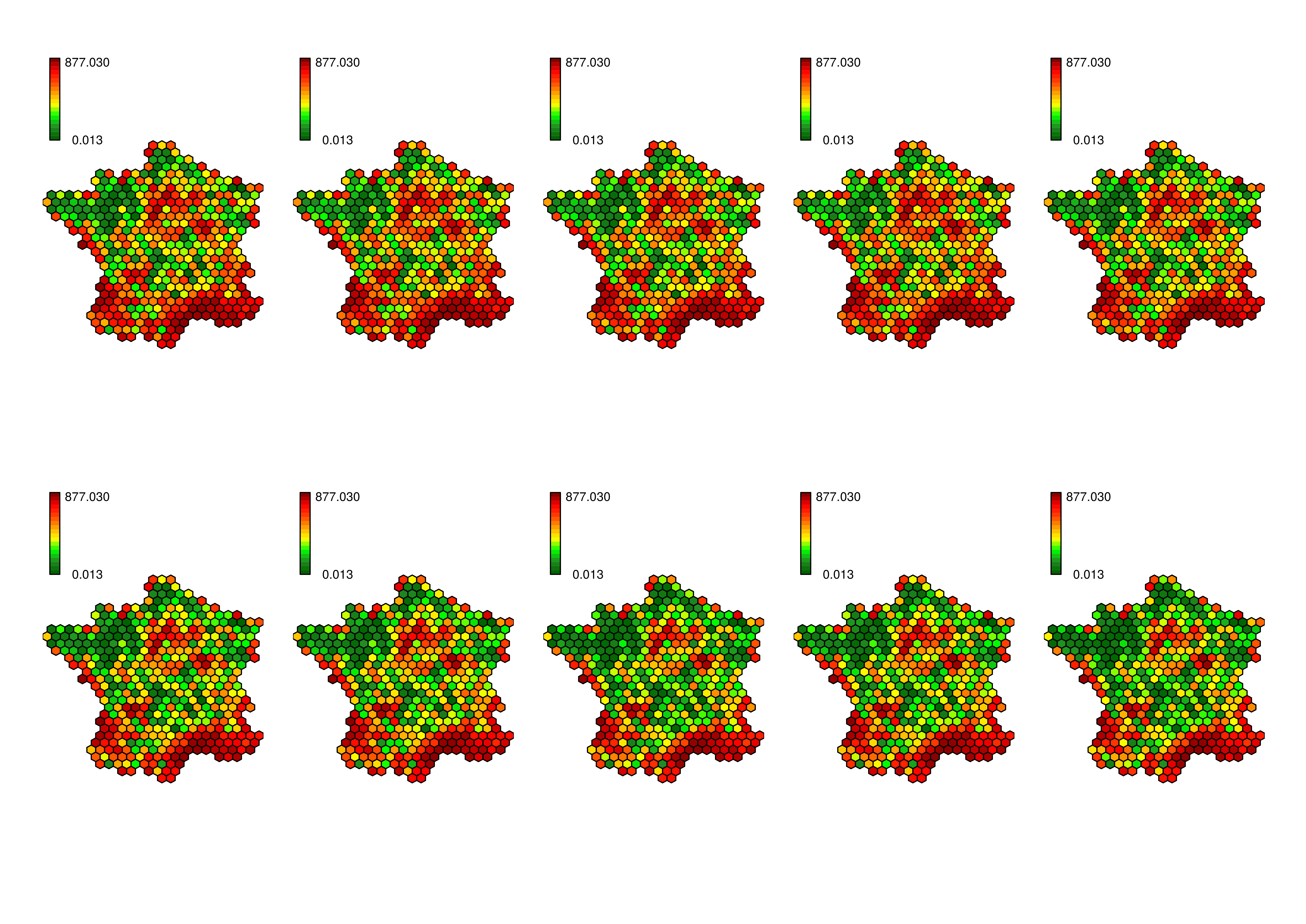

Supplement: S6 Fig — (TIFF) [file pone.0222898.s006.tiff]

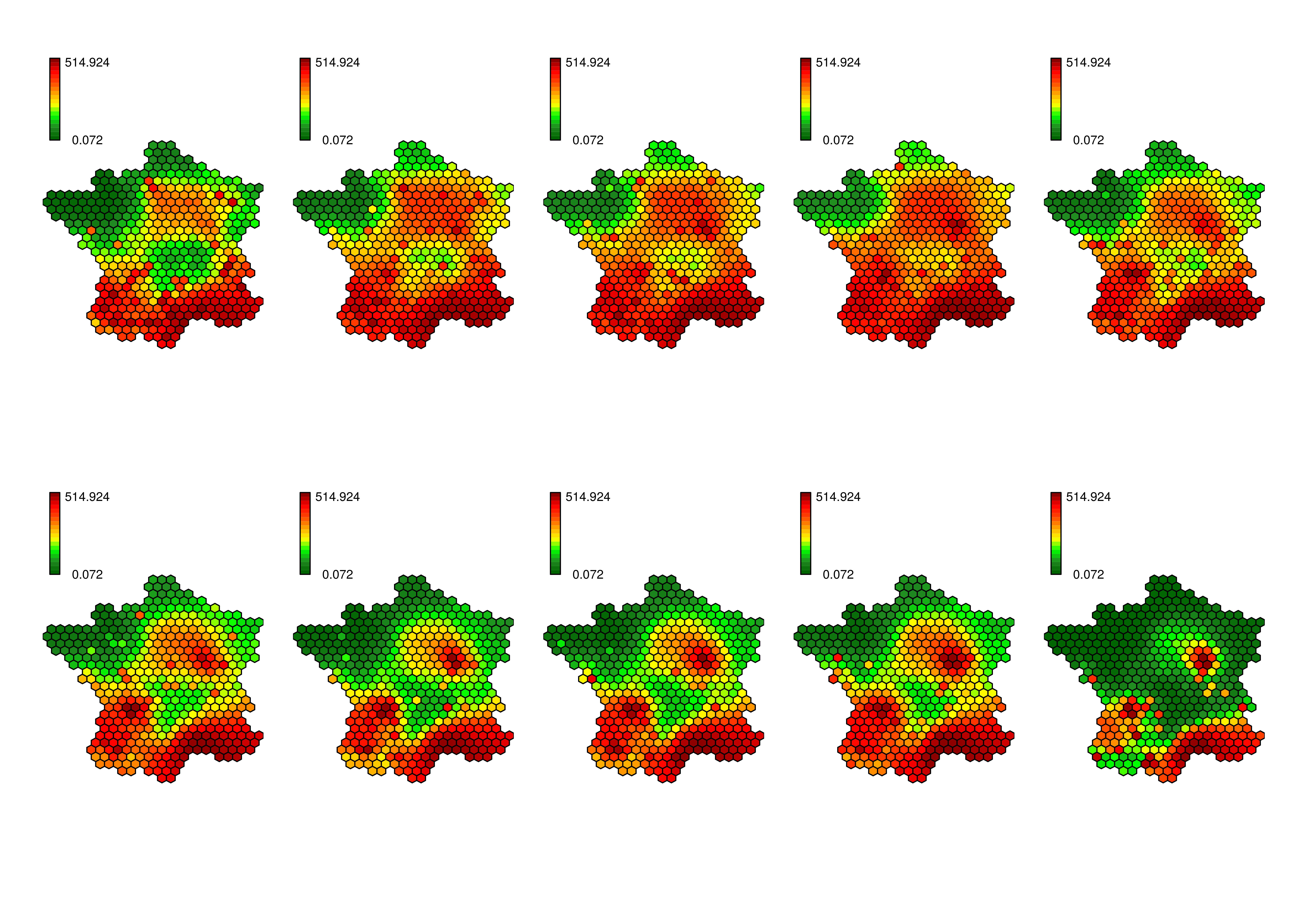

Supplement: S7 Fig — (TIFF) [file pone.0222898.s007.tiff]
