# Supplementary material for: Bayesian hierarchical models for disease mapping applied to contagious pathologies
Source: PLoS One. 2021 Jan 13;16(1):e0222898. doi: 10.1371/journal.pone.0222898 (PMC7806170; doi:10.1371/journal.pone.0222898)
Supplement: S1 Table — (PDF) [file pone.0222898.s008.pdf]

S1 **Table.** Names and characteristics of the considered models.

Name of the model	Distribution at the first level	Structure of the risk
poissnopar_x_x_xx_xxxx	Poisson	$S_{ij} = 0$
poissnopar_x_x_xx_gaus	Poisson	$S_{ij} = \epsilon_{ij}$
poissnopar_x_x_ST_xxxx	Poisson	$S_{ij} = V_{ij}$
poissnopar_x_x_ST_gaus	Poisson	$S_{ij} = V_{ij} + \epsilon_{ij}$
poissnopar_x_T_xx_xxxx	Poisson	$S_{ij} = T_{ij}$
poissnopar_x_T_xx_gaus	Poisson	$S_{ij} = T_{ij} + \epsilon_{ij}$
poissnopar_x_T_ST_xxxx	Poisson	$S_{ij} = T_{ij} + V_{ij}$
poissnopar_x_T_ST_gaus	Poisson	$S_{ij} = T_{ij} + V_{ij} + \epsilon_{ij}$
poissnopar_S_x_xx_xxxx	Poisson	$S_{ij} = U_{ij}$
poissnopar_S_x_xx_gaus	Poisson	$S_{ij} = U_{ij} + \epsilon_{ij}$
poissnopar_S_x_ST_xxxx	Poisson	$S_{ij} = U_{ij} + V_{ij}$
poissnopar_S_x_ST_gaus	Poisson	$S_{ij} = U_{ij} + V_{ij} + \epsilon_{ij}$
poissnopar_S_T_xx_xxxx	Poisson	$S_{ij} = U_{ij} + T_{ij}$
poissnopar_S_T_xx_gaus	Poisson	$S_{ij} = U_{ij} + T_{ij} + \epsilon_{ij}$
poissnopar_S_T_ST_xxxx	Poisson	$S_{ij} = U_{ij} + T_{ij} + V_{ij}$
poissnopar_S_T_ST_gaus	Poisson	$S_{ij} = U_{ij} + T_{ij} + V_{ij} + \epsilon_{ij}$
poissparam_x_x_ST_xxxx	Poisson	$S_{ij} = c.V_{ij}$
poissparam_x_x_ST_gaus	Poisson	$S_{ij} = c.V_{ij} + \epsilon_{ij}$
poissparam_x_T_xx_xxxx	Poisson	$S_{ij} = b.T_{ij}$
poissparam_x_T_xx_gaus	Poisson	$S_{ij} = b.T_{ij} + \epsilon_{ij}$
poissparam_x_T_ST_xxxx	Poisson	$S_{ij} = b.T_{ij} + c.V_{ij}$
poissparam_x_T_ST_gaus	Poisson	$S_{ij} = b.T_{ij} + c.V_{ij} + \epsilon_{ij}$
poissparam_S_x_xx_xxxx	Poisson	$S_{ij} = a.U_{ij}$
poissparam_S_x_xx_gaus	Poisson	$S_{ij} = a.U_{ij} + \epsilon_{ij}$
poissparam_S_x_ST_xxxx	Poisson	$S_{ij} = a.U_{ij} + c.V_{ij}$
poissparam_S_x_ST_gaus	Poisson	$S_{ij} = a.U_{ij} + c.V_{ij} + \epsilon_{ij}$
poissparam_S_T_xx_xxxx	Poisson	$S_{ij} = a.U_{ij} + b.T_{ij}$
poissparam_S_T_xx_gaus	Poisson	$S_{ij} = a.U_{ij} + b.T_{ij} + \epsilon_{ij}$
poissparam_S_T_ST_xxxx	Poisson	$S_{ij} = a.U_{ij} + b.T_{ij} + c.V_{ij}$
poissparam_S_T_ST_gaus	Poisson	$S_{ij} = a.U_{ij} + b.T_{ij} + c.V_{ij} + \epsilon_{ij}$
nebinnopar_x_x_xx_xxxx	Negative Binomial	$S_{ij} = 0$
nebinnopar_x_x_xx_gaus	Negative Binomial	$S_{ij} = \epsilon_{ij}$
nebinnopar_x_x_ST_xxxx	Negative Binomial	$S_{ij} = V_{ij}$
nebinnopar_x_x_ST_gaus	Negative Binomial	$S_{ij} = V_{ij} + \epsilon_{ij}$
nebinnopar_x_T_xx_xxxx	Negative Binomial	$S_{ij} = T_{ij}$
nebinnopar_x_T_xx_gaus	Negative Binomial	$S_{ij} = T_{ij} + \epsilon_{ij}$
nebinnopar_x_T_ST_xxxx	Negative Binomial	$S_{ij} = T_{ij} + V_{ij}$
nebinnopar_x_T_ST_gaus	Negative Binomial	$S_{ij} = T_{ij} + V_{ij} + \epsilon_{ij}$
nebinnopar_S_x_xx_xxxx	Negative Binomial	$S_{ij} = U_{ij}$
nebinnopar_S_x_xx_gaus	Negative Binomial	$S_{ij} = U_{ij} + \epsilon_{ij}$
nebinnopar_S_x_ST_xxxx	Negative Binomial	$S_{ij} = U_{ij} + V_{ij}$
nebinnopar_S_x_ST_gaus	Negative Binomial	$S_{ij} = U_{ij} + V_{ij} + \epsilon_{ij}$
nebinnopar_S_T_xx_xxxx	Negative Binomial	$S_{ij} = U_{ij} + T_{ij}$
nebinnopar_S_T_xx_gaus	Negative Binomial	$S_{ij} = U_{ij} + T_{ij} + \epsilon_{ij}$
nebinnopar_S_T_ST_xxxx	Negative Binomial	$S_{ij} = U_{ij} + T_{ij} + V_{ij}$
nebinnopar_S_T_ST_gaus	Negative Binomial	$S_{ij} = U_{ij} + T_{ij} + V_{ij} + \epsilon_{ij}$
nebinparam_x_x_ST_xxxx	Negative Binomial	$S_{ij} = c.V_{ij}$
nebinparam_x_x_ST_gaus	Negative Binomial	$S_{ij} = c.V_{ij} + \epsilon_{ij}$
nebinparam_x_T_xx_xxxx	Negative Binomial	$S_{ij} = b.T_{ij}$
nebinparam_x_T_xx_gaus	Negative Binomial	$S_{ij} = b.T_{ij} + \epsilon_{ij}$
nebinparam_x_T_ST_xxxx	Negative Binomial	$S_{ij} = b.T_{ij} + c.V_{ij}$
nebinparam_x_T_ST_gaus	Negative Binomial	$S_{ij} = b.T_{ij} + c.V_{ij} + \epsilon_{ij}$
nebinparam_S_x_xx_xxxx	Negative Binomial	$S_{ij} = a.U_{ij}$
nebinparam_S_x_xx_gaus	Negative Binomial	$S_{ij} = a.U_{ij} + \epsilon_{ij}$
nebinparam_S_x_ST_xxxx	Negative Binomial	$S_{ij} = a.U_{ij} + c.V_{ij}$
nebinparam_S_x_ST_gaus	Negative Binomial	$S_{ij} = a.U_{ij} + c.V_{ij} + \epsilon_{ij}$
nebinparam_S_T_xx_xxxx	Negative Binomial	$S_{ij} = a.U_{ij} + b.T_{ij}$
nebinparam_S_T_xx_gaus	Negative Binomial	$S_{ij} = a.U_{ij} + b.T_{ij} + \epsilon_{ij}$
nebinparam_S_T_ST_xxxx	Negative Binomial	$S_{ij} = a.U_{ij} + b.T_{ij} + c.V_{ij}$
nebinparam_S_T_ST_gaus	Negative Binomial	$S_{ij} = a.U_{ij} + b.T_{ij} + c.V_{ij} + \epsilon_{ij}$
