# Supplementary material for: Bayesian hierarchical models for disease mapping applied to contagious pathologies
Source: PLoS One. 2021 Jan 13;16(1):e0222898. doi: 10.1371/journal.pone.0222898 (PMC7806170; doi:10.1371/journal.pone.0222898)
Supplement: S2 Table — (PDF) [file pone.0222898.s009.pdf]

S2 **Table.** Ranking of the models for the bovine tuberculosis data.

Ranking	Model	DIC value
1	nebinparam_S_T_ST_xxxx	2680.4
2	nebinparam_x_T_ST_gaus	2690.6
3	nebinparam_x_T_ST_xxxx	2700.1
4	nebinparam_S_T_xx_gaus	2710.6
5	nebinparam_x_T_xx_xxxx	2711.1
6	nebinparam_S_T_xx_xxxx	2731.1
7	nebinparam_x_T_xx_gaus	2736.4
8	nebinnopar_S_T_xx_xxxx	2826.8
9	nebinnopar_x_T_xx_xxxx	2836.0
10	nebinparam_S_T_ST_gaus	2876.1
11	nebinnopar_x_T_xx_gaus	2886.9
12	nebinnopar_S_T_ST_gaus	2895.1
13	nebinnopar_x_T_ST_xxxx	2939.4
14	nebinnopar_S_T_xx_gaus	2960.8
15	poissnopar_S_T_ST_xxxx	2975.3
16	poissnopar_S_T_ST_gaus	2975.9
17	poissnopar_S_T_xx_xxxx	2977.0
18	poissnopar_x_T_xx_gaus	2980.2
19	poissnopar_S_T_xx_gaus	2980.4
20	nebinnopar_x_T_ST_gaus	2985.0
21	poissnopar_x_T_ST_xxxx	2994.5
22	poissnopar_x_T_xx_xxxx	2999.5
23	poissnopar_x_T_ST_gaus	3012.4
24	nebinnopar_S_T_ST_xxxx	3026.7
25	nebinnopar_S_x_ST_xxxx	3186.8
26	poissnopar_S_x_ST_gaus	3191.2
27	nebinnopar_x_x_ST_gaus	3192.6
28	nebinnopar_x_x_ST_xxxx	3197.1
29	poissnopar_S_x_ST_xxxx	3199.0
30	poissnopar_x_x_ST_gaus	3210.5
31	poissnopar_x_x_ST_xxxx	3211.8
32	nebinnopar_S_x_ST_gaus	3239.2
33	nebinparam_S_x_ST_xxxx	3240.8
34	nebinnopar_S_x_xx_gaus	3245.7
35	nebinnopar_S_x_xx_xxxx	3256.3
36	poissnopar_S_x_xx_gaus	3272.8
37	nebinparam_S_x_ST_gaus	3274.2
38	poissnopar_S_x_xx_xxxx	3274.8
39	nebinparam_x_x_ST_gaus	3374.2
40	nebinparam_S_x_xx_xxxx	3411.1
41	nebinparam_x_x_ST_xxxx	3462.0
42	nebinparam_S_x_xx_gaus	3515.2
43	nebinnopar_x_x_xx_gaus	3650.0
44	poissnopar_x_x_xx_gaus	3674.7
45	nebinnopar_x_x_xx_xxxx	3832.1
46	poissparam_S_T_xx_xxxx	$+\infty$
47	poissparam_S_T_xx_gaus	$+\infty$
