# Supplementary material for: Bayesian hierarchical models for disease mapping applied to contagious pathologies
Source: PLoS One. 2021 Jan 13;16(1):e0222898. doi: 10.1371/journal.pone.0222898 (PMC7806170; doi:10.1371/journal.pone.0222898)
Supplement: S1 Code — (PDF) [file pone.0222898.s010.pdf]

S1 Code. In order to allow to reproduce the simulation study, the R codes `simulate data sets.R` and `simulation results analyse.cpp` are given separately. The RData `carte_hexa_448.RData` contains geometrical informations necessary to draw the maps and the population.

In order to apply this methodology on other real datasets, we provide codes concerning the analysis of the BUGS results : a complete code (`real_data_set_analyse.R`) and and a focus on the important parts of computing the risk and calculating the DIC (`risk_and_DIC.r`). Complete examples of the BUGS models are also given in `BUGS_models.txt`. This file contains a complete model for a Negative Binomial distribution and another for the Poisson distribution, including spatial, temporal and spatio-temporal CAR components as well as the corresponding weights. They can be easily adapted for simpler models removing unnecessary elements. When a map of geometrical units is avalaible in R (e.g. carte in `carte_hexa_448.RData` file), neighborhood can built with the poly2nb(carte) function.
