# Supplementary material for: Bayesian hierarchical models for disease mapping applied to contagious pathologies
Source: PLoS One. 2021 Jan 13;16(1):e0222898. doi: 10.1371/journal.pone.0222898 (PMC7806170; doi:10.1371/journal.pone.0222898)
Supplement: Attachment — Submitted filename: ReviewerComments.pdf [file pone.0222898.s017.pdf]

Review PONE-D-19-25032

Comments to the authors

The manuscript by Dr. Coly et al. presents Bayesian hierarchical models for disease mapping with a particular focus on infectious diseases. To be more precise, Dr Coly presents the conditional autoregressive models, which are often used in the context of chronic diseases. They argue that this class of models can be used for infectious diseases since secondary occurrences of these diseases are expected to be close in time and space. Motivated by the bovine tuberculosis data during 2001-2010 in France, they simulate data based on existence of overdispersion and varying risk differences and fit a series of Bayesian hierarchical models varying their distribution (Poisson or negative binomial), including random effects (spatial, temporal and spatiotemporal) and some weights capturing the contribution of the random effects. They compare model performance using the deviance information criterion (DIC). Based on the simulation and the DICs, they conclude that the negative binomial models handle better locally overdispersed and/or strongly structured data and additionally apply their model selection to bovine tuberculosis in France. Their resulting maps are in line with the literature highlighting regions such as Dordogne, Cote-d'Or, Chamargue and Southwest, which are known to be of higher risk. Overall it is a well and carefully written paper, scientifically solid that gives a nice description of tools already used in the literature. A downside of the paper is that neither the methods presented nor the application are novel. The simulation makes it stand from the literature, however the use of the DIC as the sole performance criterion makes it poor and complicates the interpretation. Nevertheless, the study presents results of original research, sufficiently explained and without overstatements.

Major comments

1. Page 10, Simulated data. The are certain things that can be improved in the way this section is reported and conducted. My first suggestion is to put all the information explained in details as supporting information. Things that are missing include:

   - "We simulated in each dataset 3 disease outbreaks which were characterized by a circular increase of the risk". This statement is rather vague and more information is needed with respect to the radius of the circular increase and the maximum risk increase in

the centre of the radius. How were these values selected? Is this in line with known bovine tuberculosis outbreaks? How are the results expected to change while these values change?

- More clarification is also needed to explain the 3 outbreak scenarios. To be precise, information is needed on how the hotspot moved in space over time (O2), and how the hotspot increased or decreased over time (O3: the magnitude of increase/decrease and the time trend selected).
- It is not clear how the outbreak scenarios (O_1, O_2, O_3) were combined with the COLR, COSR, NOLR, NOSR. All the possible combinations are expected to give 12 scenarios but it seems that this is not the case. Can you provide more information?

My second suggestion include the simulation metrics. DIC, although widely used, seems like a poor choice when the truth is known. Perhaps an error term quantifying differences between the true and fitted risk would give a more objective answer. In addition to this, when simulating from a Poisson, one expects the Poisson models to perform better, and when from a negative binomial, the negative binomial ones to perform better. Of course you have added some scenarios of outbreak (which is not very clear how the were combined, and perhaps this leads to this comment), but it seems to me that using the DIC, the simulation study argues towards the efficacy of DIC to retrieve the true model (and whether it overfits or not).

2. Page 20, Paragraph 3: "Thus it seems relevant to study... the smoothing of estimated risk." This is a very important sentence and needs further discussion or even a paragraph. Can the authors provide more information and recommendations on "relevant combinations of criteria or new criteria"? What is in the literature that could be used instead?

Minor comments

1. Page 2, paragraph 2: "Since its introduction, ... or overdispersion, etc.". The authors should provide citation with examples of the aforementioned advances.
2. Page 3, paragraph 3: "In the context of infectious diseases, ... of the primary case." I agree with the authors' statement, although it can be considered as a naive approach. Information about sexual behaviour, working place or city connectivity might play a stronger role in the

spread of an infection rather than a distance or neighbourhood based proximity. The authors should add a sentence acknowledging this.

3. Page 6, paragraph 2: "Such a spatiotemporal interaction implies a strong". It seems that a word is missing here after the word strong.

4. Page 6, Formula. The indexes differ from the ones on page 5. In addition, how did you deal with potential identifiability issues between the Gaussian term and the space-time interaction?

5. Page 8, Paragraph 2: "Some studies show that the criterion... have been identified". Can you provide some citations supporting this?

6. Page 11, comments about INLA. The statement about negative binomial and INLA is wrong. Negative binomial is specified inside the inla call writing `family = "nbinomial"`. In addition, the statement that "INLA was quite new and not of common use at the beginning of this study" is a bit awkward since INLA was introduced 11 years ago.

7. Table 2. How did you select which models to show on Table 2?

8. Page 12, line 4. There is a typo on the word "structur".

9. Page 18. The selection of `nebinopa_S_x_ST_xxxx` and the justification raises questions:

   - It seems weird that the temporal trend (which is a constant for all regions in a specific time point) leads to such area specific fluctuations. Can the authors also provide the same maps (on the supporting information) for `nebinopa_S_T_ST_xxxx` and `nebipara_S_x_ST_xxxx` to understand the influence of the weights?
   - The authors should also tone down the overfitting argumentation, since the truth in this case is unknown. Statements as "the risk is not spatially smooth enough" and "overfitting tendency of DIC" are rather vague and not supported by the data and results in hand.

10. Page 20 Paragraph 2: "We also remarked that our method overestimates the real... (and non-contagious) data." I do not see which part of the results support this statement.

11. Page 20. The last paragraph seems a bit off topic. I thus suggest to remove, or formulate it as avenues for future research.

12. Figures 1, 3, 8 and 9. It is hard to read the values on the colourkey. I thus suggest to make the numbers bigger.

13. Figures 4-6. The inclusion of Gaussian noise for COSR and COLR leads to a fluctuation on the DIC not seen on the cases without the Gaussian noise. Can the authors provide some interpretation of this behaviour on the results.

14. Supporting information. The only supporting information available was Fig 10 and Fig 11, not being in line with the table of contents of the supporting information on page 21.
