# Supplementary material for: Bayesian hierarchical models for disease mapping applied to contagious pathologies
Source: PLoS One. 2021 Jan 13;16(1):e0222898. doi: 10.1371/journal.pone.0222898 (PMC7806170; doi:10.1371/journal.pone.0222898)
Supplement: Attachment — Submitted filename: Response_to_reviewers.pdf [file pone.0222898.s018.pdf]

Drs Garrido Myriam & Coly Sylvain
UMR EPIA
INRAE Centre Auvergne-Rhône-Alpes
Site de Theix
63122 Saint Genès Champanelle

Editorial Board
PLOS ONE

Saint Genès Champanelle, February 17, 2020,

Dear editorial manager,

As you aware, we have submitted our manuscript *Bayesian hierarchical models for disease mapping applied to contagious pathologies* to PLOS ONE in September 2019. We have received the review (n°PONE-D-19-25032) and your revision request on January 9, 2020, and we intend to answer it through this rebuttal letter and the revised (marked and unmarked) versions of the manuscript.

We thank the reviewers for their interest and their positive advice on the manuscript. Their comments are interesting and raise relevant questions. We have edited our paper to respond to their concerns. In particular, we have clarified and provided some details about the description of the three outbreak scenarios, and also added some references to justify our statements concerning the Deviance Information Criterion (DIC). The points raised during the review process are addressed hereafter.

Preliminary comment: Some methodological choices have been deservedly discussed by the reviewers, especially concerning the model comparison and the inference of the parameters. The main aim of our paper is to determine the most efficient models for infectious data. We have primarily chosen the DIC to compare models and the Bayesian inference using BUGS because these are the most used methods in the literature. Despite the potential relevance of alternative methods, we intended to make our study as standard as possible (concerning model selection, hyperparameter distributions, and parameter inference) in order to keep the focus on the best way to model infectious data in the disease mapping framework. It seemed to us that making unusual methodological choices may have weakened the main results.

Reviewer 1: We agree that the DIC has been designed to compare slightly different and/or nested models, according to its originator Spiegelhalter. That’s why we have considered a complete diagram to handle this issue: each combination of the considered parameters (Poisson and Negative Binomial distributions, different CAR processes, Gaussian noise, weights) has been tested, resulting in 60 different models.

Alternative methods to the DIC have been proposed: BPIC [Ando], DIC* [Gelman], $DIC_{2\text{-}8}$ [Celeux], WAIC [Watanabe], RDIC. However most of them do not show any real improvement over the DIC and thus are rarely used, or they are not suitable in our context. In particular the WAIC [Watanabe, 2010] is known as an efficient model comparison method in a Bayesian framework, but

the disease cases should be separated into independent subsets [Gelman, 2014]: that is not possible in our study as all the cases are spatially and temporally dependent.

Reviewer 2:

Major 1 (page 10): The structure of the *Simulated data* section has been changed and we have clarified the description of the outbreaks. Maps have also been added in the supporting information to clarify the behavior of each outbreak. We have provided technical details to answer the concerns raised by the reviewers. In particular:

- the radius of the outbreaks (O1, O2, O3) has been chosen congruently with the size of the bovine tuberculosis outbreaks in France which concern 3 to dozens of hexagons;
- the speed of the moving outbreak (O1) is chosen to cover a large area: in particular it crosses regions with many farms (Northwest: Bretagne) and others with very few farms (Southeast) to in order to challenge the detection ability of the models;
- the outbreak (O3) increases geometrically from 2001 (no outbreak) to 2005 (maximal outbreak similar to (O1) and (O2)) then decreases geometrically from 2006 (maximal outbreak) to 2010 (no outbreak) ;
- the parameters of the negative binomial distribution are chosen so that the overdispersion value is 5.5, congruently with bovine tuberculosis real data;
- the ratio between minimal and maximal risk values is defined as 16 for the LR (= Large Risk differences) scenarios and as 4 for the SR (= Slight Risk differences) scenarios.

The 3 outbreaks (O1, O2, O3) are common to all the datasets. For each of the 4 scenarios (NOLR, NOSR, COLR, COSR), 100 datasets have been simulated, resulting in 400 datasets. We believe that the modifications performed in this section clarify these points.

Concerning the second suggestion, we agree the reviewers that considering a goodness of fit criterion based on an error term is very appealing. As the real risk is available, we have considered the MSE to compare models. However, as pointed out in the results (page 18) and in the conclusion (page 20) the estimated risk can dramatically increase and reach unrealistic values (sometimes over 200) in sparsely populated areas. In such cases, the MSE values are strongly influenced by these extreme values and fail to provide a relevant ranking of the models. As a conclusion, comparison criteria based on the errors between real and fitted risk values do not seem very efficient to evaluate the ability of the models to detect the simulated outbreaks.

« *… when simulating from a Poisson, one expects the Poisson models to perform better, and when from a negative binomial, the negative binomial ones to perform better* ». Contrary to this expectation, it appears that the negative binomial distribution provides better results for the scenario NOLR even if data are simulated with a Poisson distribution. This could be explained by the fact that when the parameter of the Poisson distribution is very different from an area to another, overdispersion occurs. In this case, the local overdispersion of the negative binomial distribution could partly handle the combination of the variability of the risks and of the heterogeneous repartition of the population.

« *but it seems to me that using the DIC, the simulation study argues towards the efficacy of DIC to retrieve the true model (and whether it overfits or not).* » We aware the DIC limitations, that’s why

we mention it sometimes in our paper. However this criterion enabled us to select relevant models to detect the simulated outbreaks and to handle both the overdispersion and spatiotemporal structure of the data.

Major 2 (page 20, paragraph 3): Like the reviewers, we think that model comparison in the disease mapping framework is a very interesting issue. We have added some sentences to provide more details about it. Indeed we plan further work on this topic. We have considered many criteria alternative to the DIC whether the real risk is known (MSE, MAE, Spearman or Pearson correlations of the risk, ...) or not (non-penalized deviance, estimated number of parameters, DIC*, DIC_2, ...). However all these approaches failed to provide readable maps, even when the true risk is known to be spatially smooth. We have also considered combinations of these methods. This is under investigation. It seems us relevant to combine a deviance based criterion and an index ensuring that the resulting risk is smooth enough to provide actionable information for the epidemiologists.

Minor 1 (page 2, paragraph 2): References have been added to illustrate the different advances in the disease mapping framework.

Minor 2 (page 3, paragraph 3): We understand that this approach can be seen as a little naive. In fact in a human epidemiology context, some factors such as sexual behavior and population flows surely play a stronger role in contamination processes rather than a distance or neighborhood based proximity. However these transmission means are disease specific and we intend to make our study as general as possible. In animal epidemiology, the disease generally spreads from one farm to another because of the contamination of certain wild animal species. In this context, the outcome of a case increases the outcome of secondary cases in the neighborhood. Few sentences have been added to clarify it and to enable other propagation means.

Minor 3 (page 6, paragraph 2): The sentence has been corrected.

Minor 4 (page 6, formula): The indexings have been corrected. Some notations have also been modified.

We have not encountered issues concerning the identifiability between the Gaussian term and the space-time interaction. In our mind the space-time interaction term is meant to handle the spatiotemporal spread of the disease while the Gaussian noise describes the random outcome of primary cases. Moreover the addition of a Gaussian noise in the models including a space-time interaction term implies very different repartitions of the risk, so the two terms seem to play a different role in our study.

Minor 5 (page 8, paragraph 2): Citations have been added to justify this statement.

Minor 6 (page 11): We agree with the reviewers that our explanation for not using INLA methods is a bit weak and dated. At the beginning of the computations (2015) on which our study is based, the negative binomial distribution and the combination of several CAR processes were not available in R packages, even if it changed then. Moreover a very few studies in a spatiotemporal context and/or in epidemiology had been performed. However these arguments are no longer applicable. The main reason we used the Bayesian inference with BUGS is that we wanted to make classical choices to keep focused on modeling the spatiotemporal correlations and the overdispersion due to the infection.

We have modified this paragraph to clarify our objectives and avoid any dated statement concerning the INLA methods.

Minor 7 (table 2): We have listed all the models for which the DIC does not exceed the DIC of the best model by more than 10. This value has been chosen congruently with the recommendations of Spiegelhalter [2002, 2004]. Spiegelhalter considers a difference greater than 10 as significant. A sentence has been added to precise this point.

Minor 8 (page 12, line 4). The error has been corrected.

Minor 9 (page 18). We have rewritten some sentences in the section about the interpretation of the results:

• We understand the surprise of the reviewer at this observation; however there is absolutely no doubt that for bovine tuberculosis data, the temporal CAR process leads to such area specific fluctuations. Maps very similar to nebi_nopa_S_T_ST_xxxx are obtained for all the 30 models including the temporal CAR, whether they include weights or not. The influence of the weights on the resulting maps is less obvious as these maps are very different from each other. However the models including the weights are ranked higher on average. For each model, the estimated weight values are different from each other and are different from 1. We can nevertheless notice that including weights fail to reduce the influence of the temporal CAR term and thus to counterbalance the geographic heterogeneity of the risk.

• Some studies mention an overfitting behavior of the DIC [Celeux 2006, Gelman 2014]. In our study, the maps selected as the best ones according to the DIC are difficult to interpret and no epidemiological conclusions about bovine tuberculosis can be drawn. However we agree with the reviewer that this issue may not necessarily be related to the DIC. Thus we have rewritten this paragraph to tone down our conclusions.

Minor 10 (page 20, paragraph 2): *"We also remarked that our method overestimates the real... (and non-contagious) data."* This statement is based on the interpretation of the bovine tuberculosis results (page 18, paragraph between figures 8 and 9): the risk is high around the Camargue and in the neighborhood of Paris despite the very few number of farms in these regions.

Minor 11 (page 20): The influence of the shape of the area in which data are aggregated appears us as an interesting question but we agree the reviewers that the last paragraph is a bit off topic. We have removed this last paragraph and added few sentences in the Materials and Methods section to explain our choice (hexagonal areas).

Minor 12 (figures 1, 3, 8 and 9): The numbers have been enlarged to improve the readability of the colourkey.

Minor 13 (figures 4-6): As remarked by the reviewers, the inclusion of Gaussian noise for COSR and COLR leads to a fluctuation on the DIC compared to the cases without the Gaussian noise. A possible explanation to this dispersion of DIC values is that sometimes the overdispersion can be rightly handled by the noise, sometimes absolutely not, depending on the random outcome of the primary cases. However this statement is just a supposition. Identifiability issues between the Gaussian noise and the spatiotemporal interaction term do not appear as a valid hypothesis to us. In fact the spatiotemporal CAR process seems to decrease the variability induced by the Gaussian noise (see figures 4 and 6).

Minor 14: Some references issues have been solved so that the support information should be now congruent with the table of contents.

We have addressed the editorial issues concerning the figures, the layout and the support information.

We have also provided R and BUGS scripts which have been used for our analyses, including data simulation, BUGS analyses, DIC computations and results operatings.

We believe that the manuscript is now suitable for publication in PLOS ONE. We stand at your disposal for any further questions or comments.

Drs Garrido Myriam and Coly Sylvain.

On behalf of all authors.
